# A Novel Microbicide/Contraceptive Intravaginal Ring Protects Macaque Genital Mucosa against SHIV-RT Infection *Ex Vivo*

**DOI:** 10.1371/journal.pone.0159332

**Published:** 2016-07-18

**Authors:** Guillermo Villegas, Giulia Calenda, Shweta Ugaonkar, Shimin Zhang, Larisa Kizima, Olga Mizenina, Agegnehu Gettie, James Blanchard, Michael L. Cooney, Melissa Robbiani, José A. Fernández-Romero, Thomas M. Zydowsky, Natalia Teleshova

**Affiliations:** 1 Population Council, New York, New York, United States of America; 2 Aaron Diamond AIDS Research Center, Rockefeller University, New York, New York, United States of America; 3 Tulane National Primate Research Center, Tulane University, Covington, Louisiana, United States of America; Centers for Disease Control and Prevention, UNITED STATES

## Abstract

Women need multipurpose prevention products (MPTs) that protect against sexually transmitted infections (STIs) and provide contraception. The Population Council has developed a prototype intravaginal ring (IVR) releasing the non-nucleoside reverse transcriptase inhibitor (NNRTI) MIV-150 (**M**), zinc acetate (**Z**A), carrageenan (**C**G) and levonorgestrel (**L**NG) (**MZCL IVR**) to protect against HIV, HSV-2, HPV and unintended pregnancy. Our objective was to evaluate the anti-SHIV-RT activity of MZCL IVR in genital mucosa. First, macaque vaginal tissues were challenged with SHIV-RT in the presence of (i) MIV-150 ± LNG or (ii) vaginal fluids (VF); available from studies completed earlier) collected at various time points post insertion of MZCL and MZC IVRs. Then, (iii) MZCL IVRs (vs. LNG IVRs) were inserted in non-Depo Provera-treated macaques for 24h and VF, genital biopsies, and blood were collected and tissues were challenged with SHIV-RT. Infection was monitored with one step SIV *gag* qRT-PCR or p27 ELISA. MIV-150 (LCMS/MS, RIA), LNG (RIA) and CG (ELISA) were measured in different compartments. Log-normal generalized mixed linear models were used for analysis. LNG did not affect the anti-SHIV-RT activity of MIV-150 *in vitro*. MIV-150 in VF from MZC/MZCL IVR-treated macaques inhibited SHIV-RT in vaginal mucosa in a dose-dependent manner (p<0.05). MIV-150 in vaginal tissue from MZCL IVR-treated animals inhibited *ex vivo* infection relative to baseline (96%; p<0.0001) and post LNG IVR group (90%, p<0.001). No MIV-150 dose-dependent protection was observed, likely because of high MIV-150 concentrations in all vaginal tissue samples. In cervical tissue, MIV-150 inhibited infection vs. baseline (99%; p<0.05). No cervical tissue was available for MIV-150 measurement. Exposure to LNG IVR did not change tissue infection level. These observations support further development of MZCL IVR as a multipurpose prevention technology to improve women’s sexual and reproductive health.

## Introduction

Millions of women worldwide need protection from sexually transmitted infections (STIs), in particular HIV, and methods to prevent unintended pregnancies. In 2013, there were 2.1 million new HIV infections and 1.5 million deaths due to HIV/AIDS [1]. Women account for about half of HIV infections, with the majority of infections transmitted via sexual intercourse [2]. Worldwide, ~38% of all pregnancies, or approximately 80 million, are unintended, with half of these pregnancies ending in abortion [3]. One in ten pregnancies ends in unsafe abortions, leading to maternal disabilities and deaths [3].

We developed an intravaginal ring (IVR) releasing the NNRTI MIV-150, zinc acetate dihydrate, carrageenan, and levonorgestrel (MZCL). Unlike other multipurpose prevention technology (MPT) IVRs in development, dapivirine/LNG (HIV/unintended pregnancy), vicriviroc/progestin (HIV/unintended pregnancy) and tenofovir/LNG (HIV/HSV-2/unintended pregnancy) [4–7], our MZCL IVR contains antiviral agents targeting HIV (MIV-150, zinc acetate), HSV-2 (CG and CG with zinc acetate), and HPV (CG) [8–15]. There is a concern for the potential emergence of drug-resistant HIV following use of microbicides containing antiretroviral drugs in infected individuals who are either unaware of their HIV infection status or who are aware but still choose to use the microbicide. The MIV-150 and ZA combination provides anti-HIV-1 activity, increasing potency and minimizing drug resistance issues [12]. In agreement with these data, topical delivery of MIV-150 from IVR for 56d in rhesus macaques led to emergence of one drug resistance mutation (E138K, associated with intermediate or low-level resistance to NNRTIs) in 2 out of 6 animals (430 RT sequences analyzed total; 0.46%), indicating a low probability for the emergence of drug resistance mutations after sustained topical delivery of MIV-150 [16].

No other MPTs being developed target HPV, and while vaccines are available, they have accessibility and cost issues and only protect against specific HPV types [17, 18]. The preclinical testing of MZC gel showed that it is safe and highly effective against immunodeficiency viruses (explants and macaques), HSV-2 (mice) and HPV (mice) [10, 12–15]. Phase 1 testing of MZC gel (PC-1005) demonstrated that vaginally applied gel (once daily for up to 14 days) is safe, well tolerated and results in low MIV-150 absorption. Moreover, cervicovaginal lavages taken 4h after dosing had significant anti-HIV and anti-HPV activity [19].

LNG, a licensed contraceptive that has been approved for non-oral controlled-release delivery via implants and intrauterine systems (IUSs), is included in several microbicide/contraceptive IVRs under development [4–7]. However, only limited data on the immunomodulatory effects of LNG are available [20–22] and it is unknown whether exposure to LNG can increase the risk of HIV transmission [23].

It has been previously shown that prototype core-matrix macaque-sized MZC and MZCL IVRs released all APIs *in vitro* for at least 90d and *in vivo* for at least 28d [24]. Moreover, MIV-150 in blood and vaginal fluids (VF) and LNG in blood reached levels associated with efficacy (protection against vaginal SHIV-RT challenge and contraceptive activity)[16, 25–28]; CG was detected in VF [24].

This study is an extension of the earlier prototype IVRs studies [24] and was designed to evaluate the anti-SHIV-RT activity of MZCL IVRs in genital mucosa. First, we determined whether LNG interferes with MIV-150’s anti-HIV activity. Then activity of VF from macaques carrying MZC or MZCL IVRs (available from previously completed studies) [24] was tested. Finally, we determined whether vaginal and cervical mucosae from animals administered MZCL IVRs (vs. LNG IVRs) are protected against SHIV-RT soon (24h) after IVRs insertion and then related protective effects to MIV-150 concentrations in tissues, VF and plasma.

## Materials and Methods

### Ethics statement

Adult female Chinese and Indian rhesus macaques (*Macaca mulatta*) were housed and cared for in compliance with the regulations under the Animal Welfare Act [29], the Guide for the Care and Use of Laboratory Animals [30], at Tulane National Primate Research Center (TNPRC; Covington, LA). All studies were approved by the Animal Care and Use Committee of the TNPRC (OLAW assurance #A4499-01). Animals were monitored continuously by veterinarians to ensure their welfare. Animals in this study were socially housed unless restricted by study design. Housing restrictions were scientifically justified and approved by the IACUC as part of protocol review. Animals were housed indoors in climate controlled conditions with a 12/12 light/dark cycle. Animals in this study were fed commercially prepared monkey chow twice daily. Supplemental foods were provided in the form of fruit, vegetables, and foraging treats as part of the TNPRC environmental enrichment program. Water was available at all times through an automatic watering system. The TNPRC environmental enrichment program is reviewed and approved by the IACUC semiannually. Extensive efforts are made to find compatible pairs for every study group, with additional environmental enrichment of housing space through a variety of food supplements and physical complexity of the environment. A team of 11 behavioral scientists monitors the wellbeing of the animals and provides direct support to minimize stress during the study period. Anesthesia was administered prior to and during all procedures, and analgesics were provided afterwards as previously described [12, 25]. All biopsy procedures were performed by Board Certified veterinarians (American College of Laboratory Animal Medicine). Biopsies were collected not more often than every 4 weeks. Veterinarians at the TNPRC Division of Veterinary Medicine have established procedures to minimize pain and distress through several means. One animal got sick during *in vivo* IVR (APIs concentrations and tissue PD study) and was euthanized. Vaginal necropsy tissues derived from additional animals were available from separate concurrent studies. The animals were euthanized using methods consistent with recommendations of the American Veterinary Medical Association (AVMA) Guidelines for Euthanasia. The animal is anesthetized with tiletamine/zolazepam (each at 4 mg/kg IM) and given buprenorphine (0.01 mg/kg IM) followed by an overdose of pentobarbital sodium. Death is confirmed by auscultation of the heart and pupillary dilation. TNPRC is accredited by the Association for Assessment and Accreditation of Laboratory Animal Care (AAALAC#000594).

### Macaque specimens

#### MIV-150/LNG toxicity, anti-SHIV-RT activity *in vitro* and *ex vivo* (VF PD)

Necropsy vaginal tissue specimens were available from n = 16 SHIV or SHIV/HSV-2 exposed, uninfected and infected Chinese and Indian macaques. Six of these macaques were Herpes B positive. Vaginal biopsies were collected from n = 11 Naïve, SHIV exposed, uninfected and infected Chinese and Indian macaques. One of these animals was Herpes B positive. Animal ages ranged from 5.7–22 years old and their weights ranged from 4.3–12 kg.

VF (swabs) were available from SHIV/HSV-2 exposed, uninfected and infected Chinese and Indian macaques with MZC (n = 11) and MZCL (n = 9) IVRs inserted for 21 or 28d in previously completed studies [24]. Animal ages ranged from 5.25–13.15 years old and their weights ranged from 5.5–14 kg.

#### *In vivo* IVR study (API concentrations and tissue PD)

Vaginal and cervical biopsies, blood samples and VF (swabs) were derived from n = 10 Naïve Indian macaques. Animals were inserted MZCL (n = 5) and LNG (n = 5) IVRs for 24h. Animal ages ranged from 7–12.7 years old and their weights ranged from 6–12 kg.

### Active pharmaceutical ingredients (APIs) for *in vitro* experiments

MIV-150 was developed by Medivir AB (Sweden) and licensed to the Population Council [12]. LNG was purchased from CrystalPharma (Valladolid, Spain). Gynol (toxicity control) was purchased from www.drugstore.com.

### IVRs

IVRs were prepared as described in [24]. Briefly, 20mm x 4mm IVRs consisted of an ethylene vinyl acetate 40 (EVA) matrix ring body loaded with 3 mg MIV-150/0.6 mg LNG and a solid core filled with 30 mg ZA /70 mg CG (MZCL IVR) or an EVA matrix containing 3 mg MIV-150 and 30 mg ZA/70 mg CG in the core (MZC IVR). LNG IVRs consisted of an EVA/0.6 mg LNG matrix and an empty core. A 500 μm core-side pore was drilled into the IVR to release ZA and CG.

### Viral stocks

SHIV-RT, generated and titered as described in [12, 31], was used for testing MIV-150 activity in PBMCs, activity of MIV-150 ± LNG in tissues and for VF PD studies. To prepare SHIV-RT stock for tissue PD studies, macaque PBMCs were depleted of CD8^+^ cells and activated for 6 days in the presence of 10 nM retinoic acid (RA) (Sigma Aldrich, St. Louis, MO), 20 U/ml IL2 (NCI BRB Preclinical Repository, Frederick, MD) and 50 ng/ml of anti-CD3 mAbs (clone OKT3; e-Bioscience, San Diego, CA) in complete RPMI 1640 (Cellgro Mediatech, Manassas, VA) containing 10% FBS (Life Technologies, Grand Island, NY), 100 U/ml penicillin—100 μg/ml streptomycin (Cellgro Mediatech) [32, 33]. Activated PBMCs were then challenged with 10^3^ TCID_50_ of SHIV-RT/10^6^ cells. Titration was performed in CEMx174 cells. A single SHIV-RT stock was used in tissue PD studies.

### SIV *gag* qRT-PCR

SHIV-RT infection was measured directly in 5 μl of tissue culture supernatants by a one step SIV *gag* reverse transcription quantitative PCR using KAPA SYBR FAST One-Step qRT-PCR Kit (KAPA Biosystems, Wilmington, MA) [34]. Specific SIV *gag* primers (Integrated DNA Technologies, Coralville, IA) were SIV667 *gag* (5′ GGTTGCACCCCCTATGACAT 3′) and SIV731 *gag* (5′ TGCATAGCCGCTTGATGGT 3′). Results were analyzed by the standard curve method, using SIVmac1A11 DNA obtained from Dr. Paul Luciw through the NIH AIDS Reagent Program, Division of AIDS, NIAID, NIH. Cycling conditions were: Step 1: 1× 42°C 5 min, Step 2: 1× 95°C 5 min, Step 3:40× (95°C 3 sec, 60°C 20 sec). Dissociation curves were generated to verify absence of unspecific amplification. PCRs were performed and data were analyzed using the ViiA^™^ 7 real time PCR system and software respectively (Applied Biosystems, Carlsbad, CA). The LLOQ of the assay is 10 copies/5μl. The coefficients of inter and intra-assay variability are 1.55 and 1.32 respectively.

### MIV-150 activity in PBMCs

Human PBMCs were isolated from leukopacks (NY Blood Center, New York, NY) and were activated with 3X3 stimulation method as previously described [10, 35]. MIV-150 was added to activated PBMCs (2x10^5^/well in U-bottom 96-well plates) at different concentrations (0.04–10 nM) and incubated for 1h in U-bottom 96-well plates before adding 100 TCID_50_ of SHIV-RT followed by an overnight incubation at 37°C. The supernatant was replaced with fresh stimulation media on days 1 and 4 post infection. The p24 levels were tested on day 7 after infection using the p24 ELISA (Zeptometrix, Buffalo, NY). The EC_50_ and EC_90_ values were calculated using a dose-response-inhibition analysis on GraphPad Prism v5.0 software. All MIV-150 concentrations were tested in triplicates.

### MIV-150/LNG toxicity, anti-SHIV-RT activity *in vitro* and *ex vivo* (VF PD)

#### Vaginal tissue and VF collection and processing

Necropsy vaginal tissue (at least 0.5–1cm^2^) and vaginal biopsies (n = 2 at each collection time; 3x5mm each) were utilized. Tissues (biopsies, necropsy) were transported in complete L-15 medium to our laboratory on ice overnight and then cut into 3x3mm explants using skin biopsy punches (Acu-Punch, Acuderm, Fort Lauderdale, FL) [36, 37]. Explants were pooled and randomly assigned treatments (below).

VF from macaques with MZC (n = 9) and MZCL (n = 11) IVRs were available from previously completed studies [24]. VF were collected using Merocel^®^ spears (Medtronic Xomed, Jacksonville FLA) at the baseline (right before IVR insertion), and at 4, 48, 72h and 9d after insertion and placed in saline and processed as in [38]. Aliquots of VF were stored at -80°C. VF MIV-150 concentrations were measured as described below.

#### MIV-150/LNG toxicity and anti-SHIV-RT activity *in vitro*

Viability of explants following ~18h incubation with MIV-150 and LNG (vs. gynol) was tested by MTT assay as described in [37]. To test anti-SHIV-RT activity, vaginal explants were prepared from biopsy and necropsy tissues, stimulated with 5 μg/ml PHA (Sigma Aldrich) and 100 U/ml IL2 in cDMEM for 48h [36]. Then, tissues were challenged with 10^4^ TCID_50_ SHIV-RT per explant in the presence of MIV-150 (1.5 and 0.15 μM), LNG (6 and 0.6 μM) alone or in combination (three explants per condition). After ~18h tissues were washed and cultured in the presence of IL2 for 14d. Infection (pooled replicates) was monitored by one step SIV *gag* qRT-PCR (as above) on tissue culture supernatants collected at days 0 (post last wash), 3, 7, 11 and 14 [34]. SOFT and cumulative (CUM) analyses (d3-14 as d0 represents carryover input virus) were performed as described in [36, 39, 40].

#### VF PD

Vaginal explants were prepared from biopsies and necropsies as above. Anti-SHIV-RT activity of MIV-150 released in macaque VF was tested in vaginal explants as described in [36]. Briefly, PHA/IL2 stimulated explants were challenged with SHIV-RT as above in the presence of 1:5 diluted VF (two-three explants per condition). Then tissues were washed and cultured in cDMEM containing IL2. Infection (pooled replicates) was monitored by RETRO-TEK SIV p27 Antigen ELISA (ZeptoMetrix, Buffalo, NY). p27 SOFT and CUM endpoint analyses (d3-14) were performed as above.

### *In vivo* IVR study (APIs concentrations and tissue PD)

MZCL (n = 5) and LNG (n = 5) IVRs were inserted in non-Depo Provera (non-Depo)-treated macaques for 24h. Vaginal swabs were collected immediately before IVR removal, placed in PBS/1%FBS and processed as described in [38] and stored at -80°C until assayed for MIV-150 and CG concentrations. Blood was collected immediately before IVR removal. Serum LNG, plasma MIV-150, VF MIV-150 and VF CG concentrations post IVR removal were measured as below.

Vaginal (n = 2 per time point; 3x5mm each) and cervical (n = 2 per time point; 3x4.5mm each) [36] biopsies were collected 24h post IVR insertion and at the baseline (performed 5 weeks after IVR removal). One animal in the LNG group developed recto-vaginal fistula and was euthanized. Therefore, the data include n = 5 and n = 4 animals in MZCL and LNG groups, respectively. In 2 out of 4 animals in the LNG group, ectocervical biopsies were not available. Tissues were processed immediately for *ex vivo* challenge as described below. A portion of vaginal biopsies (5–21 mg) collected post IVR insertion was kept at -80°C until assayed for MIV-150 concentration.

#### Tissue PD study

Anti-SHIV-RT activity of tissue-associated MIV-150 was determined in biopsies collected 24h post MZCL and LNG IVRs insertion (vs. baseline). Biopsies were rinsed with PBS, cut into 3x3mm explants using skin biopsy punches as described above within an hour of collection and challenged with 10^4^ TCID_50_ SHIV-RT per explant (2–6 vaginal and 2–4 cervical explants) in the presence of 100 U/ml IL2. ~18h after challenge, tissues were washed and cultured in cDMEM containing IL2 for 14d [36]. Supernatants were collected at d0 (after washes), 3, 7, 11 and 14d of culture. Infection was monitored by one step SIV *gag* qRT-PCR and SIV *gag* SOFT and CUM endpoint analyses (d3-14) were performed as above.

### MIV-150, LNG and CG quantification

MIV-150 was measured by LCMS/MS in plasma (LLOQ = 20 pg/ml) [13] and in tissues (LLOQ = 0.025 ng/mg) [41], and by RIA in VF (LLOQ = 1 ng/ml) [12]. LNG was measured by RIA in serum (LLOQ = 47 pg/ml) (Oregon National Primate Research Center (ETSC, Beaverton, OR). CG was measured by ELISA in VF (LLOQ = 40 ng/ml) [41].

### Statistics

#### MIV-150/LNG toxicity

Analysis of the toxicity experiments was done using a log-normal generalized linear mixed model. OD_570_ was the response variable, treatment was the predictor. Animal and animal-treatment interactions were included as random effects.

#### MIV-150/LNG activity against tissue SHIV-RT infection *in vitro*

SOFT and CUM p27 values (pooled replicate values) were analyzed using log-normal generalized linear mixed models with treatment as the only predictor. A random intercept for each animal was included. Pairwise tests were performed with Tukey-adjusted *t* tests.

#### VF PD

Anti-SHIV-RT activity of MIV-150 released in VF (vs. baseline VF) was analyzed using a log-normal generalized linear mixed model to predict the SOFT and CUM p27 values (pooled replicate values). The treatment (MZC or MZCL IVRs) and the log-transformed MIV-150 concentrations were the predictors. Both the biopsy animal ID and the VF animal ID were included as random effects.

#### Tissue PD

For CUM and SOFT analyses, SIV *gag* copy number of individual replicate values ≥ LLOQ were assumed log-normal. Any value <LLOQ (2000 copies/ml) at 3-14d of culture was set to $2000^{1/\sqrt{2}}=215.86$, a common substitution for log-normal data. CUM from 3-14d for replicates below LLOQ corresponds to 863.44 copies/ml. Comparisons of CUM and SOFT S*IV gag* copies endpoints were performed using a log-normal generalized linear mixed model. The treatment (MZCL vs. LNG IVRs), biopsy time (baseline vs. post IVR exposure) and their interactions were used a predictors. A random intercept and time were added as random effects within an animal ID. Overall significance was determined by the Type 3 *F*-test, and pairwise comparisons were made with Tukey-adjusted *t* tests. To analyze if inhibition of SHIV-RT infection in the tissue is dependent on the concentration of MIV-150 in VF, plasma, or vaginal tissue, these concentrations were used separately as predictors of SOFT and CUM results in log-normal generalized linear mixed models. All models included a random intercept for each animal ID.

## Results

### LNG does not alter MIV-150 activity against *ex vivo* SHIV-RT challenge in macaque vaginal explants

**Fig 1:**
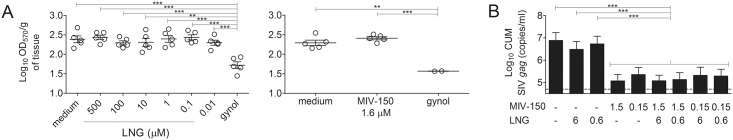
LNG does not affect the activity of MIV-150 (non-toxic concentrations) in macaque vaginal mucosa. (**A**) Macaque vaginal explants were immersed in culture media containing LNG or MIV-150 (vs. 1:10 diluted gynol) for ~18h. Tissue viability was determined using MTT assay (OD_570_ of the formazan product was measured in triplicates and normalized to the dry weight of the explants). Each symbol indicates an individual donor and the Mean±SEM of the Log_10_ OD_570_/g of tissue for each condition is shown. (**B**) PHA/IL2 stimulated explants were challenged with SHIV-RT (10^4^ TCID_50_/explant; triplicates) in the presence of 1.5 or 0.15 μM MIV-150 and/or 6 or 0.6 μM LNG (vs. untreated control). 18h later, tissues were washed and cultured for 14d with IL2 and infection was monitored by SIV *gag* qRT-PCR in tissue supernatants. Summaries of Log_10_ CUM analyses (d3-14 of culture) of 7 experiments (Mean±SEM) are shown. Input SIV *gag* copy numbers (Mean; post washout after challenge) are shown as dotted lines. Significant *p*-values of <0.0001(***) and <0.001(**) are indicated.

To explore if LNG interferes with the antiviral activity of MIV-150, PHA/IL2 stimulated vaginal explants were challenged with 10^4^ TCID_50_ of SHIV-RT in the presence of MIV-150 ± LNG overnight (~18h). The lowest dose of 0.15 μM (50 ng/ml) MIV-150 reflects the approximate plateau level detected in VF from MZC/MZCL IVR-treated animals [24]. LNG doses of 0.6–6 μM (200–2000 ng/ml) correspond to *in vitro* daily release of LNG from MZCL IVRs [24]. Overnight incubation with MIV-150 (1.6 μM) and LNG (up to 500 μM) did not decrease explants viability as measured by MTT assay (Fig 1A). LNG at 0.6 μM and 6 μM did not change tissue infection level (Fig 1B). We previously demonstrated potent activity of unformulated MIV-150 at 0.16 μM (the lowest concentration tested) against SHIV-RT in macaque vaginal explants [14]. 0.15–1.5 μM MIV-150 inhibited SHIV-RT infection (vs. untreated control) and LNG doses up to 6 μM did not change activity of MIV-150 (SOFT/CUM, p>0.05). No significant differences were observed between MIV-150—LNG and MIV-150 + LNG conditions (Fig 1B). As a reference, activity of MIV-150 against SHIV-RT in PBMCs was evaluated. MIV-150 inhibited infection in PBMCs with EC_50_ = 0.32 nM and EC_90_ = 1.84 nM.

### MIV-150 in VF collected from animals administered MZC and MZCL IVRs inhibits *ex vivo* SHIV-RT infection of macaque vaginal tissue in a dose-dependent manner

**Fig 2:**
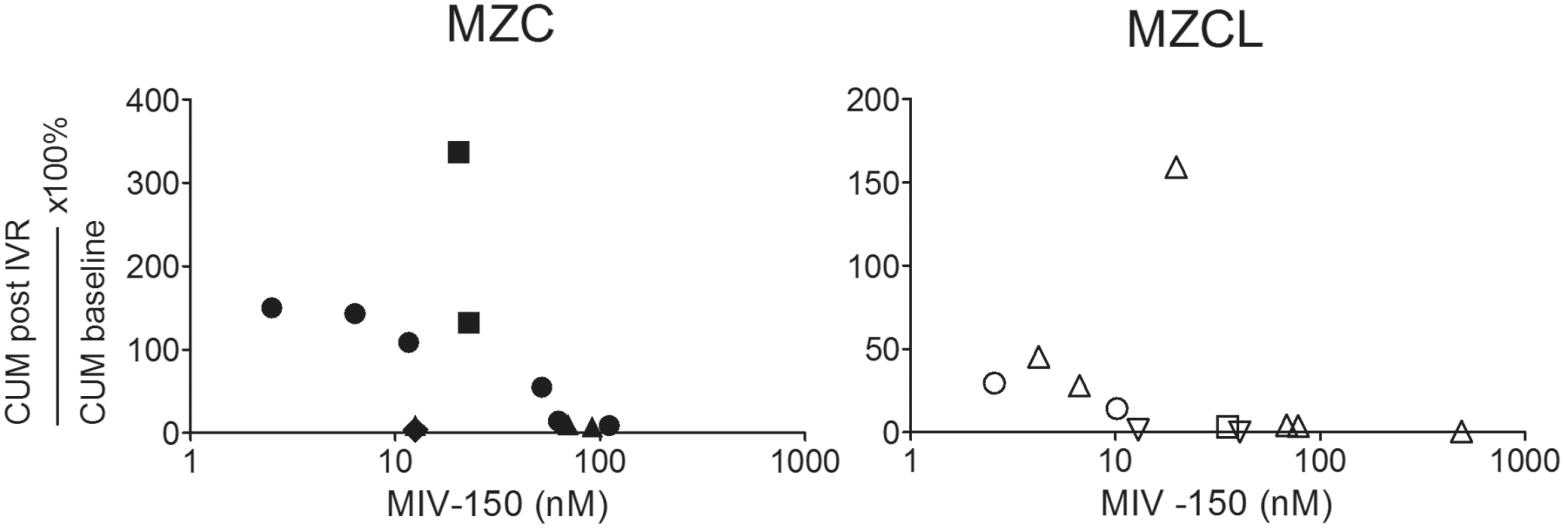
MIV-150 in VF inhibits SHIV-RT infection in vaginal mucosa in a dose-dependent manner. PHA/IL2-stimulated explants from untreated animals were challenged with 10^4^ TCID_50_ SHIV-RT/explant (duplicates/triplicates) in the presence of 1:5 diluted VF collected at the baseline and after MZC or MZCL IVR insertion at 4h, 48h, 72h and 9d (post IVR). The concentrations of MIV-150 correspond to 1:5 diluted VF. The explants were cultured for 14d in the presence of IL2. Infection was monitored by p27 ELISA (d0-14 of culture). The activity of each baseline IVR and post IVR VF pair was tested in explants from different donors represented by different symbols. Changes in CUM analyses (d3-14 of culture) in the presence of post IVR VF are shown relative to MIV-150 concentrations in diluted VF.

Anti-SHIV-RT activity of VF containing *in vivo* released MIV-150 from MZCL and MZC IVRs was tested *ex vivo*. PHA/IL2 stimulated vaginal explants were challenged with 10^4^ TCID_50_ of SHIV-RT in the presence of 1:5 diluted VF collected before and 4, 48, 72h and 9d after IVR insertion. We previously demonstrated that VF do not affect the viability of macaque vaginal explants under these experimental conditions [36]. The 1:5 diluted VF contained 2.5–490 nM of MIV-150 (MZC and MZCL IVR groups combined). MIV-150 in VF from MZC and MZCL IVRs provided dose-dependent inhibition of SHIV-RT in vaginal tissue (combined IVR groups; SOFT/CUM p = 0.02/0.01) (Fig 2). Each time the MIV-150 concentration doubled, SOFT/CUM decreased by 39%/43%. There was not enough power to detect an association between MIV-150 in VF and protection analyzing one IVR group at a time, except for MZC IVR CUM analysis (CUM p = 0.01 for MZC IVR group; p>0.05 for MZCL IVR group). No significant difference in VF activity between the two IVRs was found for CUM, while VF from MZCL IVR-carrying animals was significantly more effective than VF from MZC IVR animals for SOFT (p = 0.04). Therefore, LNG does not decrease the activity of MIV-150 in our models. Much like we observed with the MIV-150 IVR, where ≥19 nM MIV-150 in the VF strongly inhibited SHIV-RT infection [36], >20 nM MIV-150 in the VF from animals inserted MZCL IVRs strongly reduced infection.

### Tissue-associated MIV-150 inhibits *ex vivo* SHIV-RT infection

**Fig 3:**
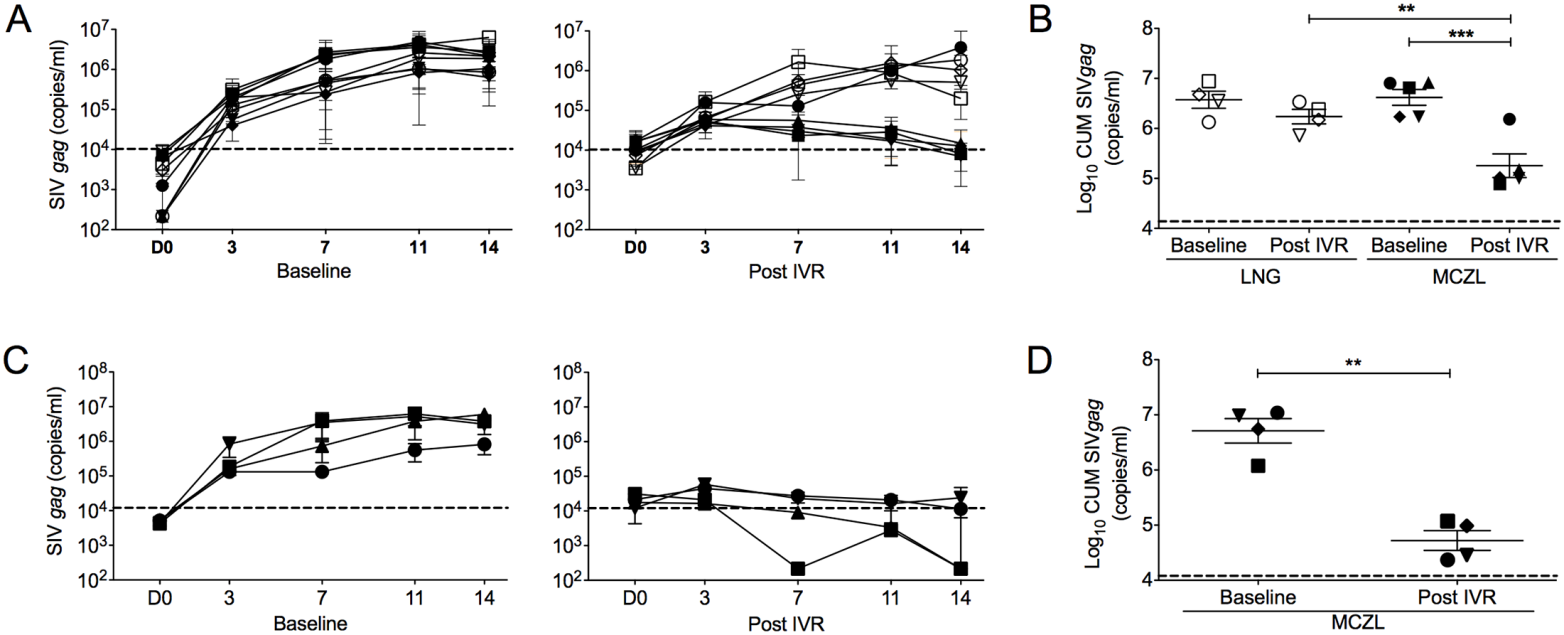
Tissue-associated MIV-150 inhibits *ex vivo* SHIV-RT infection of vaginal and cervical mucosa. MZCL (n = 5) or LNG (n = 4) IVRs were inserted for 24h. Vaginal and ectocervical biopsies were collected immediately after IVR removal (Post) and at the baseline. Non-stimulated vaginal (n = 2–6) and cervical (n = 2–4) explants were challenged with SHIV-RT (10^4^ TCID_50_/explant) for ~18h, washed and cultured for 14d with IL2. Infection was monitored and analyzed as in Fig 1. Shown are (**A**) SIV *gag* copies/ml of each animal (Mean of replicates ± SEM; symbols match those shown in panel B) and (**B**) SIV *gag* CUM analyses of Log_10_ transformed data (Mean ± SEM) in vaginal tissues. Each symbol represents an individual animal (MZCL group: closed symbols; LNG group: open symbols). Shown are (**C**) SIV *gag* copies/ml of each animal (Mean of replicates ± SEM; symbols match those shown in panel D) and (**D**) SIV *gag* CUM analyses of Log_10_ transformed data (Mean ± SEM) in cervical tissues. Each symbol represents an individual animal (MZCL group). Significant *p*-values of <0.0001(***) and <0.001(**) are indicated. Input SIV *gag* copy numbers (Mean; post washout after challenge) are shown as dotted lines.

Activity of tissue-associated MIV-150 was determined in vaginal and cervical biopsies 24h post IVRs insertion, at the peak of MIV-150 detected in VF, as shown in the earlier study using identical MZCL IVRs [24]. Non-stimulated vaginal (n = 2–6) and cervical (n = 2–4) explants were processed for viral challenge within one hour of collection. Baseline vaginal tissue SHIV-RT infection levels were not different between IVR groups (Fig 3A and 3B). Tissue infection post LNG IVRs exposure was comparable to infection at the corresponding baseline and to the MZCL group baseline (Fig 3A and 3B). In contrast, infection of vaginal tissues post MZCL IVRs exposure was significantly reduced relative to the respective baseline (96% inhibition, SOFT/CUM both p< 0.0001) and the post LNG IVR group (~ 90% inhibition, SOFT/CUM; p = 0.0002/0.0007) (Fig 3A and 3B). Infection of cervical tissue in the post MZCL IVR group was significantly inhibited relative to baseline (99% inhibition; SOFT/CUM p = 0.01/0.01) (Fig 3C and 3D). An insufficient number of ectocervical samples in the LNG IVR group precluded statistical analysis.

### APIs concentrations/PD assessment

MIV-150 (VF, vaginal tissues and plasma) and LNG (serum) were measured 24h post MZCL IVR insertion (Table 1). Cervical tissues were not available for assessment of MIV-150 concentrations. The levels of MIV-150 and LNG in blood, and MIV-150 in VF were comparable to those seen in earlier pharmacokinetics (PK) studies [24]. As expected based on previous data [24], CG was not detected in VF at this time. An average of 20.9 ng/mg of MIV-150 was detected in vaginal tissue (Table 1). We did not observe dose-dependent inhibition of SHIV-RT infection in vaginal tissue (S1 Fig). MIV-150 concentration in VF did not predict anti-SHIV-RT activity in the vaginal and cervical tissues (S1 and S2 Figs). Also, plasma MIV-150 concentrations did not predict anti-SHIV-RT activity in cervical tissues (S2 Fig). Of note, higher MIV-150 concentrations in plasma corresponded to higher vaginal tissue infection level (SOFT/CUM; p = 0.03/0.03). However, this result was driven by only one animal, which had the lowest tissue and the highest plasma MIV-150 concentrations (S1 Fig).

**Table 1:** MIV-150 and LNG concentrations in different compartments 24h post IVR insertion.

IVR	Animal ID	MIV-150			LNG
		VF	Plasma	Vag tissue	Serum
		(ng/ml)	(ng/ml)	(ng/mg)	(pg/ml)
**LNG IVR**	FH61	nd	nd	nd	2020
	DK37	nd	nd	0.0291	3180
	GV86	nd	nd	0.0359	4637
	EM09	nd	nd	0.0301	4376
**MZCL IVR**	ED86	104.944	0.4862	51.2548	3480
	EJ67	87.615	0.3795	8.1234	3413
	EJ98	359.434	0.3524	31.1456	4376
	EN78	282.67	0.4801	6.9605	3761
	FC79	320.066	0.5392	6.8829	4035

nd—not detected

## Discussion

Our study demonstrates that MIV-150 released from the MZC/MZCL IVRs *in vivo* (VF and/or tissue-associated) is highly effective against *ex vivo* SHIV-RT infection in vaginal and cervical mucosa. LNG did not compromise MIV-150 activity. In fact, SOFT (but not CUM) analysis revealed that VF from MZCL IVR animals more strongly inhibited SHIV-RT relative to VF from MZC IVR animals. MIV-150 in VF from the prototype MZC and MZCL IVRs provided dose-dependent inhibition of *ex vivo* SHIV-RT infection in macaque vaginal explants. MIV-150 potently inhibits SHIV-RT in PBMCs. >20 nM MIV-150 in the VF from MZCL IVRs (>10 fold EC_90_ in cell-based assays) strongly reduced infection, pointing to higher concentration of MIV-150 needed for SHIV-RT inhibition in explants vs. cell-based assays. This data are consistent with our previous report [36]. The results agree with our earlier reports documenting the activity of MIV-150 in cells and explants in the presence of VF [25, 36].

Previous PK and efficacy studies of MZC gel demonstrated that tissue MIV-150 concentrations following gel application better predict efficacy against vaginal SHIV challenge than plasma or VF MIV-150 concentrations [12]. Similarly, the NNRTI dapivirine showed concentration-dependent inhibition of HIV in cervical tissue post IVR exposure [42]. Tenofovir (TFV) concentrations in vaginal lymphocytes post gel application in macaques predicted efficacy against vaginal SHIV challenge better than plasma TFV levels (VF levels of TFV were not measured) [43–45]. PK/PD assessments in humans identified that TFV concentrations in VF and detection of TFV in plasma (CAPRISA 004) were associated with reduced HIV infection [45, 46]. These data highlight the need to have detailed assessment of compartmental PK/PD in animal models (measuring PK and PD at the same time) to inform efficacy studies and clinical trial design.

Our study provides important insights into plasma, tissue and VF MIV-150 concentrations and tissue PD relationships. Tissue-associated MIV-150 (≥6.8 ng/mg in vaginal mucosa; not measured in cervix) afforded 96% protection against *ex vivo* SHIV-RT challenge 24h post IVR exposure vs. baseline. We did not observe MIV-150 dose-dependent protection, likely because of high MIV-150 concentrations in all vaginal tissue samples. Although only 3mg of MIV-150 was loaded in the MZCL IVRs vs. 100mg of MIV-150 in the previous MIV-150 IVR studies [25, 36], the average vaginal tissue MIV-150 concentration 24h post IVR insertion in the current study was ~210 fold higher (~21 ng/mg vs. ~0.1 ng/mg [25]). MIV-150 concentration in genital tissues was reported to be sensitive to Depo treatment, which resulted in increased tissue MIV-150 concentration over time [25]. Of note, animals in the current study received no Depo. High tissue MIV-150 concentrations detected in our study are likely the result of greater release of MIV-150 from MZCL IVRs [24] compared to MIV-150 IVRs [25].

As we discussed in [36], previous studies reporting tissue concentrations of MIV-150 [25] and tissue PD [36] suggest that ~0.6 ng/mg of MIV-150 in vaginal tissue protects against *ex vivo* SHIV-RT infection. Also, PK/efficacy studies suggested that even lower concentrations of MIV-150 (~0.1 ng/mg) could protect against *in vivo* vaginal SHIV-RT challenge when IVRs remain in place post challenge [25]. However, the *in vivo* protection was lost when IVRs were removed right before the challenge [25]. MIV-150 concentrations in VF did not predict anti-SHIV-RT activity in tissues from corresponding animals. The results were somewhat expected due to the variable VF volumes in animals. The finding that higher MIV-150 plasma concentrations corresponded to higher vaginal (but not cervical) tissue infection needs to be confirmed in a larger group of animals as this effect was driven by a single animal.

It is unlikely that CG and ZA contributed to the observed anti-SHIV-RT activity in the tissue PD studies. Similar to the recent report [24], no CG was detected in macaque VF 24h post IVR insertion. VF ZA was not measured in our study as VF ZA levels were reported below the LLOQ of the assay (15 μg/ml) in a previous study [24]. We previously demonstrated no effect of single exposure to ZA at 466 μM (102 mg/ml) on SHIV-RT infection in vaginal explants [14].

We acknowledge that the small number of animals (n = 4–5 per group) in API concentrations and tissue PD studies represents a limitation. These studies are exploratory in nature and larger, powered studies are needed to confirm these results.

We previously discussed that increased ectocervical tissue infection post-Depo treatment could have resulted in a lack of infection inhibition by MIV-150 in the ectocervix [36]. It is unknown if LNG changes mucosal immune milieu and susceptibility to HIV after short-term exposure. As signaling through steroid receptors is rapid [47] and can potentially lead to changes in mucosal susceptibility to HIV, we chose to include LNG IVR control group for MZCL IVR *in vivo* studies. Similar to short-term *in vitro* LNG exposure experiments, *in vivo* exposure to LNG in non-Depo treated animals did not change vaginal tissue infection levels. As the number of ectocervical samples in LNG group was limited, we cannot conclude whether infection in cervical mucosa was affected by LNG. Long-term exposure to LNG in LNG IUD users was reported to lead to contrasting changes in the expression of CCR5 on peripheral blood CD4 and CD8 T cells and on endometrial and cervical T cells; changes in tissue (endometrium and cervix) gene expression mediating cell homing, cell-cell signaling and immune activation [20–22]. Use of combined oral contraceptives containing LNG or insertion of Norplant leads to decrease in epithelial thickness in macaques [48, 49] and may enhance susceptibility to HIV. Furthermore, LNG was recently reported to decrease genital epithelial barrier function, induce influx of inflammatory cells to the mucosa and increase susceptibility to HSV-2, which is known to increase HIV transmission [50–53]. We acknowledge that new models involving low viral challenge dose and shorter vs. longer-term exposure to LNG would be useful to address effects of LNG on mucosal tissue susceptibility to HIV/SHIV-RT.

Overall, this study shows that prototype MZCL IVRs release active MIV-150 *in vivo* within 24h hour of insertion, the MIV-150 protects vaginal and cervical mucosa against *ex vivo* challenge with SHIV-RT, and LNG does not interfere with this activity. This study, together with safety and activity data of MZC gel against immunodeficiency viruses (explants and macaques), HSV-2 (mice) and HPV (mice) [12–15, 41], indicate that the MZCL IVR is a viable MPT IVR candidate. An MZCL IVR that simultaneously prevents the sexual transmission of HIV, HSV-2, HPV, and unintended pregnancy will have the potential to significantly reduce the worldwide incidence of HIV and improve the sexual and reproductive health of millions of women.

## Supporting Information

S1 Fig MIV-150 concentrations and vaginal tissue PD. CUM SIV *gag* copy numbers from experiments summarized in Fig 3 (MZCL IVR group; Mean±SEM) are plotted against MIV-150 concentrations in vaginal tissue, VF and plasma. Each symbol represents an individual animal matching those shown in Fig 3.
(TIF)

S2 Fig MIV-150 concentrations and ectocervical tissue PD. CUM SIV *gag* copy numbers from experiments summarized in Fig 3 (MZCL IVR group; Mean±SEM) are plotted against MIV-150 concentrations in VF and plasma. Each symbol represents an individual animal matching those shown in Fig 3.
(TIF)
