# Supplementary figures and images for: A Novel Microbicide/Contraceptive Intravaginal Ring Protects Macaque Genital Mucosa against SHIV-RT Infection Ex Vivo
Source: PLoS One. 2016 Jul 18;11(7):e0159332. doi: 10.1371/journal.pone.0159332 (PMC4948912; doi:10.1371/journal.pone.0159332)

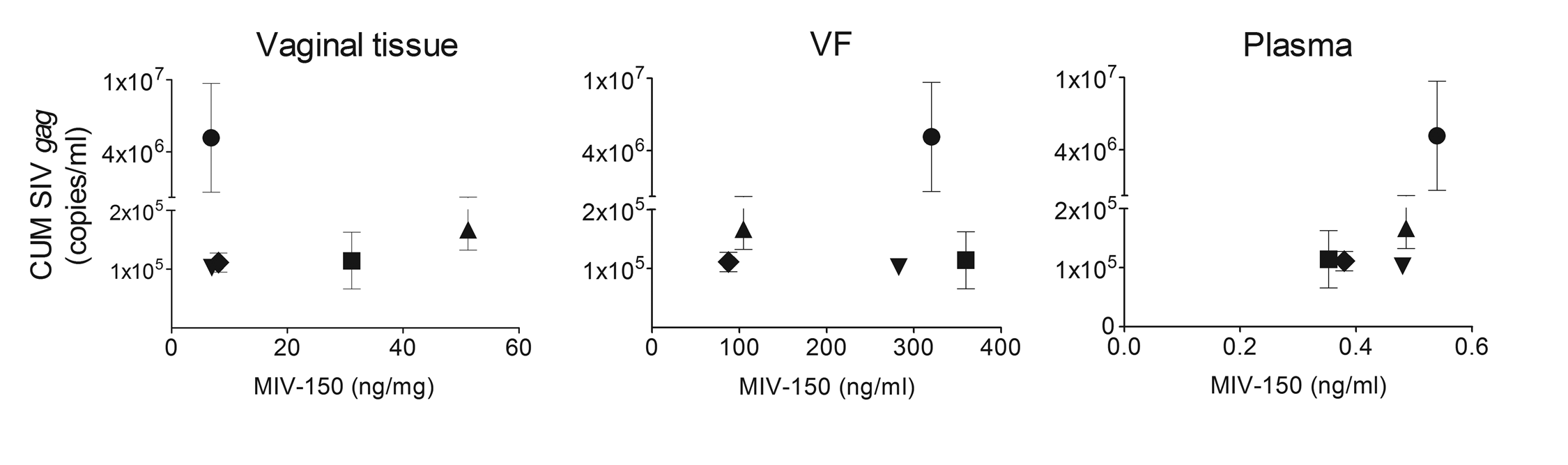

Supplement: S1 Fig — CUM SIV gag copy numbers from experiments summarized in Fig 3 (MZCL IVR group; Mean±SEM) are plotted against MIV-150 concentrations in vaginal tissue, VF and plasma. Each symbol represents an individual animal matching those shown in Fig 3. (TIF) [file pone.0159332.s001.tif]

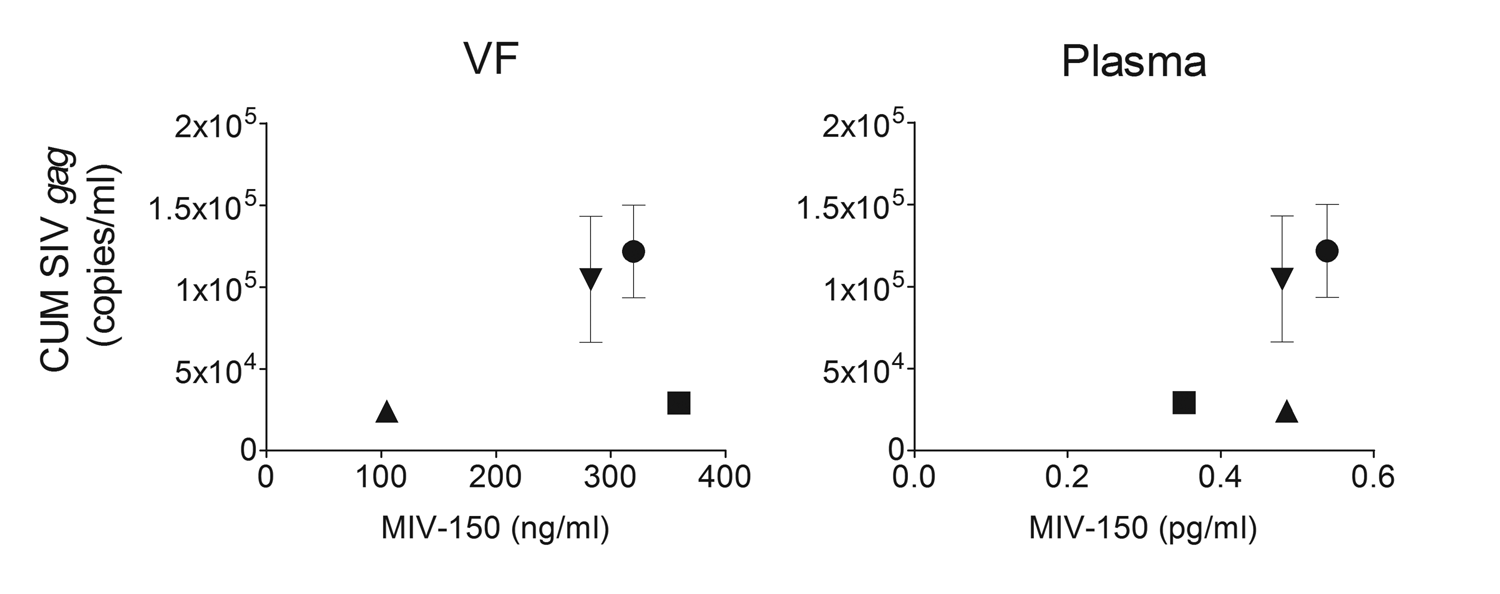

Supplement: S2 Fig — CUM SIV gag copy numbers from experiments summarized in Fig 3 (MZCL IVR group; Mean±SEM) are plotted against MIV-150 concentrations in VF and plasma. Each symbol represents an individual animal matching those shown in Fig 3. (TIF) [file pone.0159332.s002.tif]
